# Supplementary material for: Suicidal Ideation in Iraqi Medical Students Based on Research Using PHQ-9 and SSI-C
Source: Int J Environ Res Public Health. 2023 Jan 18;20(3):1795. doi: 10.3390/ijerph20031795 (PMC9914454; doi:10.3390/ijerph20031795)
Supplement: Supplementary file 1 [file ijerph-20-01795-s001.zip › ijerph-2060023-supplementary.pdf]

Table S1. College affiliation versus PHQ-9 categories.

College	PHQ-9 Category	Proportion (%)
Dentistry	Moderate Depression	28.3
	Mild Depression	27.0
	Moderately Severe Depression	18.4
	Severe Depression	15.8
	Minimal Depression	10.5
Medicine	Mild Depression	28.0
	Moderate Depression	23.3
	Severe Depression	20.3
	Moderately Severe Depression	19.1
	Minimal Depression	9.3
Pharmacy	Moderate Depression	38.0
	Mild Depression	28.7
	Moderately Severe Depression	19.4
	Severe Depression	11.1
	Minimal Depression	2.8

Table S2. Bivariate correlations for the total sample.

		Age	Gender	Marital Status	Religious Affiliations	Year of Study	Previous Psychiatric Consultation	Past History of Mental Illness	Past History of Chronic Illness	Family History of Mental Illness	Family History of Suicidality	History of Self-Medication	Recent Stress Events	PHQ-9 [total score]
Gender	Coeff.	0.035												
	Sig.	0.437												
Marital Status	Coeff.	0.167**	0.028											
	Sig.	0.000	0.528											
Religious Affiliations	Coeff.	0.047	-0.112*	-0.039										
	Sig.	0.297	0.012	0.382										
Year of Study	Coeff.	0.744**	0.095*	0.139**	0.087									
	Sig.	0.000	0.035	0.002	0.053									
Previous Psych. Consultation	Coeff.	0.120**	-0.031	0.055	0.083	0.106*								
	Sig.	0.008	0.492	0.218	0.064	0.019								
Personal History of Mental Illness	Coeff.	-0.002	0.063	0.045	.0120**	-0.016	0.347**							
	Sig.	0.964	0.163	0.313	0.007	0.724	0.000							
Personal History of Chronic Illness	Coeff.	0.029	0.063	0.061	-0.037	0.083	0.103*	0.060						
	Sig.	0.514	0.164	0.172	0.410	0.063	0.022	0.184						
Family History of Mental Illness	Coeff.	0.100*	0.021	-0.099*	0.066	0.091*	0.080	0.281**	0.025					
	Sig.	0.026	0.639	0.027	0.143	0.043	0.075	0.000	0.577					
Family History of Suicidality	Coeff.	-0.015	0.042	-0.033	0.054	0.005	0.043	0.226**	0.060	0.111*				
	Sig.	0.745	0.350	0.461	0.227	0.909	0.340	0.000	0.179	0.013				
History of Self-Medication	Coeff.	0.004	0.038	-0.078	0.042	0.054	0.123**	0.208**	0.179**	0.233**	0.192**			
	Sig.	0.925	0.398	0.084	0.356	0.226	0.006	0.000	0.000	0.000	0.000			
Recent Stress Events	Coeff.	0.121**	0.047	0.066	0.016	0.130**	0.001	0.091*	0.077	0.048	0.025	0.089*		
	Sig.	0.007	0.300	0.140	0.727	0.004	0.983	0.044	0.086	0.287	0.575	0.049		
PHQ-9 [total score]	Coeff.	0.026	0.098*	-0.022	0.070	0.030	0.103*	0.323**	0.073	0.082	0.222**	0.046	0.246**	
	Sig.	0.557	0.028	0.620	0.118	0.499	0.022	0.000	0.104	0.066	0.000	0.307	0.000	
SSI-C [total score]	Coeff.	-0.060	0.042	0.018	0.142**	-0.011	0.101*	0.323**	0.037	0.099*	0.199**	0.074	0.106*	0.554**
	Sig.	0.184	0.347	0.688	0.002	0.798	0.025	0.000	0.413	0.027	0.000	0.101	0.018	0.000

*. Correlation is significant at 0.05 level (2-tailed).

**. Correlation is significant at 0.01 level (2-tailed).

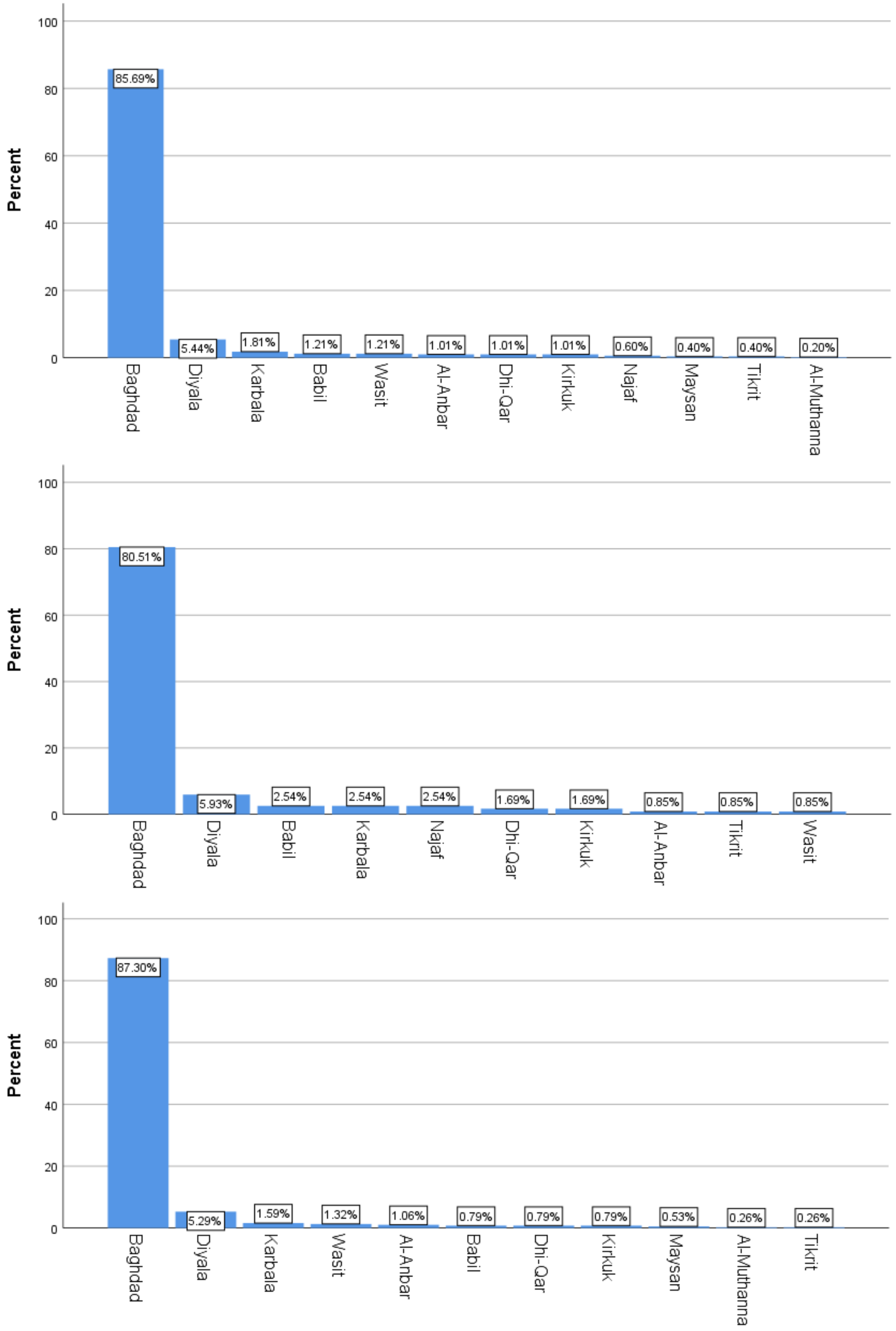

Figure S1. Place of Residence: total sample (top), males (middle), and females (bottom).
